# Current Paradigm of Hepatitis E Virus Among Pediatric and Adult Patients

**DOI:** 10.3389/fped.2021.721918

**Published:** 2021-09-30

**Authors:** Oana Belei, Oana Ancusa, Adelina Mara, Laura Olariu, Elena Amaricai, Roxana Folescu, Carmen Lacramioara Zamfir, Daniela Gurgus, Andrei G. Motoc, Livia Claudia Stânga, Liliana Strat, Otilia Marginean

**Affiliations:** ^1^First Pediatric Clinic, Disturbance of Growth and Development on Children Research Center, “Victor Babes” University of Medicine and Pharmacy, Timisoara, Romania; ^2^Fifth Department of Internal Medicine, “Victor Babes” University of Medicine and Pharmacy, Timisoara, Romania; ^3^Department of Internal Medicine, Emergency City Hospital, Timisoara, Romania; ^4^First Pediatric Clinic, “Victor Babes” University of Medicine and Pharmacy, Timisoara, Romania; ^5^Department of Rehabilitation Physical Medicine and Rheumatology, “Victor Babes” University of Medicine and Pharmacy, Timisoara, Romania; ^6^Department of Balneology, Medical Recovery and Rheumatology, Family Discipline, Center for Preventive Medicine, “Victor Babes” University of Medicine and Pharmacy, Timisoara, Romania; ^7^Department of Morpho-Functional Sciences I, “Grigore T. Popa” University of Medicine and Pharmacy, Iasi, Romania; ^8^Department of Anatomy and Embriology, “Victor Babes” University of Medicine and Pharmacy, Timisoara, Romania; ^9^Department of Microbiology, “Victor Babes” University of Medicine and Pharmacy, Timisoara, Romania; ^10^Department of Mother and Child Medicine, “Grigore T. Popa” University of Medicine and Pharmacy, Iasi, Romania

**Keywords:** hepatitis E virus, pediatric patients, adult patients, prevention, antiviral treatment

## Abstract

Hepatitis E virus (HEV) infection is a polymorphic condition, present throughout the world and involving children and adults. Multiple studies over the last decade have contributed to a better understanding of the natural evolution of this infection in various population groups, several reservoirs and transmission routes being identified. To date, acute or chronic HEV-induced hepatitis has in some cases remained underdiagnosed due to the lower accuracy of serological tests and due to the evolutionary possibility with extrahepatic manifestations. Implementation of diagnostic tests based on nucleic acid analysis has increased the detection rate of this disease. The epidemiological and clinical features of HEV hepatitis differ depending on the geographical areas studied. HEV infection is usually a self-limiting condition in immunocompetent patients, but in certain categories of vulnerable patients it can induce a sudden evolution toward acute liver failure (pregnant women) or chronicity (immunosuppressed patients, post-transplant, hematological, or malignant diseases). In acute HEV infections in most cases supportive treatment is sufficient. In patients who develop chronic hepatitis with HEV, dose reduction of immunosuppressive medication should be the first therapeutic step, especially in patients with transplant. In case of unfavorable response, the initiation of antiviral therapy is recommended. In this review, the authors summarized the essential published data related to the epidemiological, clinical, paraclinical, and therapeutic aspects of HEV infection in adult and pediatric patients.

## Introduction

The hepatitis E virus (HEV) was first characterized in detail in 1983 during an outbreak of non-A non-B, non-C hepatitis among Soviet soldiers on a military mission in Afghanistan (1). The first HEV strain from the animal kingdom was isolated from pigs in the United States (2). The phylogenetic analysis of HEV strains based on complete genomic sequencing of the virus allowed the characterization of four main genotypes (HEV-1, HEV-2, HEV-3, and HEV-4) (3).

HEV infection became an important health burden worldwide. This scourge has affected almost one third of the global population. HEV infection triggers an intricate dual disease, with divergent epidemiological and clinical features in low socioeconomic areas and developed countries (4, 5).

HEV infection causes substantial outbreak of acute hepatitis among inhabitants from developing countries, where there are favorable conditions for the spread of infection through contaminated water sources polluted from sewage (6). In the endemic areas HEV represents a major public health problem, causing a significant number of deaths. HEV contamination underline up to 70% of episodic acute viral liver infections in endemic countries (7). It is worrying that these epidemics erupt repeatedly over a certain period of time. Between 1978 and 1982 there were four outbreaks of hepatitis E in Kashmir, India, affecting over 50,000 inhabitants of which almost 1,700 died (8).

HEV genotypes 1 and 2 are waterborne transmitted and usually cause self-limiting episodes of hepatitis among individuals without comorbidities. Over 40% of all cases of fulminant liver failure are due to HEV infection (9, 10), with a mortality index of 51.9% especially among pregnant women (7, 11). A particular feature of the hepatitis E epidemic in developing countries is the increased incidence of the disease among pregnant women and the higher severity index compared to other subjects. Acute HEV infection can also occur outside endemic areas, in economically developed countries, the mode of transmission being mainly through contaminated food.

Foodborne transmission of HEV genotype 3 was reported as being responsible for the spread of infection in the United Stated of America and Europe, while genotype 4 is incriminated of maintaining the zoonosis in Asia (Taiwan and China) (12, 13).

An increasing number of recent papers described the development of chronic HEV infection progressing to end stage liver diseases mainly in immunocompromised subjects, including pediatric patients. It was also reported the possibility of HEV transmission through contaminated blood transfusions.

In developed countries, autochthonous acute hepatitis E cases can be easily confused with drug induced liver injury due to lack of awareness by physicians and the occurrence of the disease in older subjects that usually associate other comorbidities (14).

In highly industrialized areas, HEV genotypes 3 and 4 do not determine severe hepatitis among pregnant women compared to genotypes 1 and 2, spread in poor countries (15).

Although HEV infection primarily target the liver cells, several studies have shown that the HEV can multiply in other extrahepatic tissues such as the kidneys, digestive tract, spleen, placenta and neuronal cells (16). Therefore, besides the common liver injuries described, HEV may induce extrahepatic manifestations in children as well as in adults. These conditions may comprise central and peripheral nervous system alteration, hematological, renal and pancreatic disorders (17–20).

Acute HEV infection is not as rare as previously thought worldwide. Moreover, cases with chronic HEV infections have been reported more often lately, especially in adult or pediatric patients with liver comorbidities, immunological disorders or post-transplant.

## Papers Selection Methodology

The authors performed for this review a systematic literature search centered on the HEV infection topic among adult and pediatric patients. This research was conducted searching PubMed databases from 1990 to December 2020. All publications focusing etio-pathogeny, clinical and diagnostic aspects in pediatric and adult patients or data regarding prevention and treatment of HEV infection were reviewed and the essential data have been summarized in this paper.

### The History of HEV Infection

In recent decades, the natural course of HEV infection has been better understood. In 1978, hepatitis E was first identified by Dr. Mohammad S Khuroo as an outbreak of non-A, non-B hepatitis in Kashmir, India. He investigated an epidemic of jaundice that broke out in this area, in the Srinagar region of India (21). The hepatitis affected 200 villages that numbered 600,000 people, causing about 52,000 cases of hepatitis and 1,700 deaths. The investigation of that epidemic as well as subsequent epidemics and sporadic cases were performed on a period of 14 years. This led to the discovery of a non-A non-B hepatitis with enteric transmision, that had triggered repeated epidemics in Kashmir (6, 8). The disease had a self-limited evolution and had no consequences such as persistent viremia, chronic hepatitis and liver failure with cirrhosis (22). Over a period of more than 14 years, IgG antibodies have been lost in more than half of the population (23). The conclusion was that the cause of the disease was a human hepatitis virus still unknown (24). Later, in 1983, an outbreak of non-A non-B hepatitis was declared among Russian soldiers on a mission in Afghanistan (1), as described above. The epidemic from Afghanistan had epidemiological characteristics similar to those observed in Kashmir in 1978.

A member of the research team, Dr. Mikhail Balayan, a Russian physician, virologist, intentionally became infected by eating stool samples collected from Russian soldiers infected with HEV, who were deployed in Afghanistan, and then, after a period of 36 days after ingestion, the doctor contracted acute hepatitis, severe form, with hepatocytolysis and jaundice. Trough immune electron microscopy were identified small viral particles in his stool samples (25). This is how Dr. Balayan obtained the first morphological evidence for hepatitis E virions. They have been described as uncoated icosahedral particles with a “spiky” surface and 27–30 nm in diameter. HEV was first misclassified within the family *Caliciviridae*, due to its calicivirus-like structural appearance (1).

In 1990 Reyes et al. performed an incomplete cloning of the virus and then its entire genome was sequenced using bile samples extracted from laboratory-infected macaques (26). Later, in 1991, Tam et al. managed to sequence the full-length HEV genome of 7.2 kb and performed a diagnostic test based on enzymatic immunoassay (27). Due to the significant difference in the sequence of HEV from other known families of viruses, this virus was reclassified into a new family, *Hepeviridae*. An enzyme immunoassay has also been developed to detect antibodies against HEV (28). Later, in the United Stated, the first strain of HEV of animal origin was discovered and characterized, from pigs-swine HEV (2).

### HEV Main Characteristics Analysis

#### Virus Biology, Reservoir, and Transmission

HEV has a RNA genome of 7.2 kb with positive-sense, single-stranded, which is capped and polyadenylated at the 3 'and 5' end. The genome of this virus contains 3 open reading frames (ORFs). Of the entire genom, 2/3 encodes ORF1, a big multi-domain polyprotein that play a role in genomic replication. The remaining 1/3 of the genome encodes ORF2 and ORF3. These two are translated from a subgenomic RNA produced during the viral replication cycle. ORF2 encodes the capsid protein of HEV. The ORF3 protein is specific for HEV and is involved in virion output. A new ORF, ORF4, has recently been discovered in the coding region of ORF1, but seems to be limited only to HEV genotype 1 (29).

Latest studies indicate that the E virus exists in 2 forms: uncoated, naked virions, which are eliminated in the bile and feces to mediate inter-host transmission, and membrane-coated (a lipid membrane), quasienveloped virions that circulate in the blood flow to mediate the spread of the virus in the host. Although the two types of virions are infectious, the way they infect cells is different. Because they do not have classical envelope proteins, the mechanism by which they infect cells is atypical (30, 31).

HEV multiplies in the cytoplasm, with a subgenomic RNA that produces the proteins ORF2 and ORF3 and the complete genomic RNA that encodes the non-structural proteins and is used as a model for replication. The proteolytic processing mechanism does not affect the ORF1 protein as reported by the latest research in the field (32, 33). HEV multiplies in hepatocytes, but also in the small intestine, colon and lymph nodes, this being demonstrated by identifying the RNA intermediates with negative-sense (34, 35).

HEV genotypes 1 and 2 are human viruses. They are extremely endemic in many parts of the world, such as Africa, Asia, Mexico and the Middle East. The mode of transmission is achieved by contaminating the water with human feces (36). According to epidemiological studies, annually HEV-1 and HEV-2 cause about 20 million new infections, of which 3.4 million cases of acute hepatitis E and 70,000 deaths due to acute liver failure. In most areas of Asia and Africa the seroprevalence rate of anti-HEV antibodies is 10–40%, and in Egypt it reaches about 80% (37). HEV genotypes 3 and 4 are zoonotic viruses that have the ability to infect pigs and other animal species but also humans. Transmission between species takes place by consumption of HEV-contaminated food products and by direct contact with infected animals. It has also been observed that HEV can be transmitted from infected people to others through organ transplantation and blood transfusions.

HEV-1, 2, 3, and 4 genotypes can cause acute hepatitis with self-limited evolution, acute liver failure as well as neurological diseases. However, certain viral strains are associated with certain pathologies. Additional risk factors are age and pre-existing liver diseases. Genotypes 1 and 2 are more commonly associated with pancreatitis and high mortality rates in pregnant women (11, 19). Pregnant women in the third trimester have a mortality rate of up to 30% due to HEV genotype 1 infection, especially in the north of India (28). It is not known very well what are the main mechanisms that lead to pregnancy-related pathology, but one option would be the increase of the estrogen level in pregnancy. Thus, genotype 1 and 2 infection during pregnancy increases the risk of miscarriage, premature birth or stillbirth.

Impairment of other systems have also been reported, such as renal impairment (IgA nephropathy) and neurological impairment (Guillain-Barré syndrome and encephalitis) (16, 17).

Unlike genotypes 1 and 2, genotypes 3 and 4 are more commonly associated with neurological disorders. In addition, HEV-3 infection may increase the risk of acute or chronic liver failure in those patients with underlying liver disease (as hepatitis B or C virus infections). Immunosuppressed patients, such as recipients of organ transplants in immunosuppressive regimens, are at increased risk of developing chronic infections that can lead to development of fibrosis and cirrhosis of the liver (38–40).

#### HEV Epidemiology

The spread of the HEV is divided into four distinct epidemiological areas: hyperendemic, endemic, distinctive and sporadic.

##### Hyperendemic Areas

The spread of HEV genotype 1 and 2 is hyperendemic in several countries located in Central Asia (Kazakhstan, Tajikistan, and Uzbekistan), as well as South Asia (Sri Lanka, Pakistan, India, Bhutan, Bangladesh, and Nepal) or South-east Asia (Laos, Vietnam, Indonesia, Burma, Thailand, and Cambodgia) (6). Also, HEV genotype 1 and 2 is hyperendemic in east Africa (Burundi, Uganda, and Kenya), North Africa (Sudan, Tunisia, Morocco, and Algeria), and west Africa (Ivory Coast, Liberia, Nigeria, and Mali). In these areas, HEV infection has epidemic and endemic character. Also, the hyperendemic form is mainly caused by the HEV-1 and 2 genotype in Mexico (6).

##### Endemic Areas

The spread of HEV is endemic in several countries in the Middle East (the United Arab Emirates, Turkey, Saudi Arabia, Libya, Yemen, Bahrain, Oman, Iran, and Kuwait), some regions of Southeast Asia such as Singapore and several countries from South America (Ecuator, Brazil, Uruguay, and Argentina). In these regions, the E virus causes more than a quarter of all cases of sporadic acute hepatitis and fulminant hepatitis. However, in these endemic areas there are no epidemics of jaundice due to HEV infection. Hepatitis E has a major impact worldwide and has two different epidemiological patterns. In developing countries, HEV is a major health problem transmitted through contaminated water and most often occurs in young peoples, the disease being more severe in pregnant women and in patients with pre-existing liver cirrhosis (41, 42).

##### Distinctive Pattern Areas

In Egypt, HEV has different epidemiological characteristics and distinct features from other regions of the world. Seroprevalence in this region is similar to that of hepatitis A virus and the disease occurs at an young age. In pregnant women, HEV infection manifests as a mild form or even asymptomatic form of the disease. Egyptians are affected by the HEV-1 genotype, with subtypes not found in Asians (42, 43).

##### Sporadic Areas

In developed countries, indigenous hepatitis E is increasingly being diagnosed (44), the infection being determined by HEV-3 and HEV-4 genotype. Each year are reported ~50–100 new cases of hepatitis E in countries such as Germany, France, United Kingdom, and other countries from Europa in pediatric and adult patients. Epidemiological data regarding the disease seroprevalence show that these reported numbers are actually much lower than the real prevalence of the disease (45). Many infections caused by HEV remain unrecognized and thus are not tested or may be asymptomatic form or can be misclassified as drug-induced liver injury (DILI) (46).

The prevalence of HEV in developed countries differs within the same country, between regions.

For example, the seroprevalence of hepatitis E in the south of France reaches over 50% of the population, being four times higher than in the northern part of France (47), ~68,000 HEV infections being reported in this country in recent years. Such differences exist in other countries as well. In the United Kingdom, 100,000 HEV infections have been reported, with a significant difference between north and south, with a seroprevalence of 16% in the south-west of England, 12% in the rest of England and 4.6% in Scotland-Edinburgh. It is not known exactly why there is such a variation in seroprevalence. Data on the incidence of hepatitis E in developed countries are also limited, but estimates show a high incidence: 0.7% in the United States and 0.2% in the United Kingdom (48). In the United States, more than two million new HEV infections are reported each year. One reason for the discrepancy is that there are currently no FDA-approved diagnostic tests in the United States for human use, so many infections will simply be “missed.” In addition, there is evidence showing that most infections with HEV genotypes 3 and 4 are silent (49). Germany is another country where 300,000 HEV infections have been reported annually in recent years, based on specific antibodies to HEV. Cases increase strongly in number, the explanation being more frequent testing and better detection tests (36).

The National Health and Nutrition Evaluation Survey in the US conducted an analysis of the seroprevalence of HEV and showed that in 2010, the overall seroprevalence of HEV in the general population over the age of 6 years was estimated at around 6.0%—much higher than the prevalence of antibodies against hepatitis C virus (1.3%), and hepatitis B surface antigen (0.4%) but lower than the IgG seroprevalence of hepatitis A (34.8%). Using the multivariate analysis method, HEV seroprevalence was strongly correlated with increasing age. The prevalence of anti-HEV antibodies in pediatric population aged 6–19 years was 0.8% in boys and 1.1% in girls (50).

There are well-documented studies in the literature pointing that in travelers/workers returning to industrialized countries from areas of hyperendemicity have been described sporadic cases of HEV caused by type 1 and 2 genotypes, but the scale of imported HEV infection is not very well-known. A recent study made in Netherlands showed that both children and adults were dominated by autochthonous hepatitis, compared to cases of hepatitis imported from travel, while in Italy, most cases of hepatitis E were related to travel and are caused by genotype 1 (51, 52) (Table 1).

**Table 1:** Characteristics of HEV genotypes.

**HEV genotypes**	**1**	**2**	**3**	**4**
Year of discovery	1983	1986	1995	2003
Geographic distribution	Developing countries; Asia/Africa	Sub-Saharan Africa, Mexico	Developed countries; USA, Europe, New Zealand, Argentina	India, Eastern Asia (China, Taiwan, Japan, Vietnam)
Hosts	Human, pig	Human	Human, pig, other mammalian species, shellfish	Human, pig, other mammalian species

Autochthonous hepatitis E caused by HEV 3 and 4 genotypes is identical to manifestations like hepatitis E in developing countries, except that the patients in most cases are middle-aged persons or elderly people (mean age-60 years; male/female ratio > 3: 1). The majority of cases are sporadic, with no major outbreaks, although a number of small groups of infected cases have been reported, being well-documented, from a food source outbreak. Unfortunately, the source of the infection cannot be identified in most cases. In the majority of patients, the disease has a self-limited evolution, with the improvement of the clinical and biological picture within 4–6 weeks (36). In industrialized countries, hepatitis E is sometimes misdiagnosed as another common liver disease in adults: drug-induced liver injury (DILI) (14, 53).

There are 2 categories of patients, in whom the natural history and prognosis differ: children and adults with underlying chronic liver pathology who have a bad prognosis and children or adult patients who are immunosuppressed and in whom the infection often becomes chronic. A small percentage of patients have neurological symptoms and the diagnosis of hepatitis may be missed, because neurological manifestations are best expressed (54).

It has been concluded that HEV is currently probably the most common cause of acute viral hepatitis worldwide that infects one third of the world population (55, 56). Most data regarding HEV were initially presented in studies made on the adult population, but there are recent publications that highlight certain specific pediatric problems. There are not currently guidelines on HEV infection in children and its importance for pre-existing pediatric liver disease. Pediatric patients undergoing immunosuppressive therapy for any indication other than solid organ transplant, like nephrotic syndrome, autoimmune disease, inflammatory bowel disease, have an increased risk of chronic hepatitis E.

The general prevalence of acute HEV infection and the percentage of chronicity in immunocompromised children is not well-known, because HEV is rarely tested in children with increased liver enzymes (57, 58).

The recommendation of the ESPGHAN Committee is that immunocompetent children with elevated liver enzymes and/or extrahepatic impairment such as acute pancreatitis, haemolytic anemia, unknown cause of thrombocytopenia, or neurological symptoms, to be tested for HEV infection. The same recommendations are for immunocompromised children with abnormal transaminases, including those who have performed pediatric solid organ and stem cell transplants and other children on immunosuppressive therapy with biological changes without an identified cause must be tested for HEV (59).

The main epidemiological data and clinical characteristics of HEV infection in developing and developed countries are presented in Table 2.

**Table 2:** Epidemiology and clinical features of HEV infection in developing and developed countries.

**Item**	**HEV in developing countries**	**HEV in developed countries**
**Epidemiology**		
Genotypes	1 and 2	3 and 4
Source of infection	Human	Zoonotic; pigs are primary host^a^
Route of infection	Fecal-oral *via* infected water	Fecal-oral *via* infected pig meat, direct exposure, infected water
Transfusion-related infection	Yes	Yes
Seroprevalence	Low in children up to 15 years old, increases rapidly from ages 15 to 30	Steady increase throughout age groups
Incidence	Variable: 64/1,000 patient/year, Bangladesh	Variable: 3/100 patients/year, South of France; 7/1,000 patients/year, USA
	Yes; can involve thousands of cases	No; occasional small case clusters from a food point source
Attack rate	1 in 2	67–89% of those infected are asymptomatic
Person-to-person spread	Very limited	No
Seasonality	Yes; outbreaks occur at times of flooding/monsoon	No
Disease in travelers returning from areas of endemicity	Well-described	Is beginning to emerge as high-risk areas become defined
**Clinical features**		
Age of infection (yr)	15–30	>50
Sex (male/female ratio)	2:1	>3:1
Clinical course	Self-limiting hepatitis	Self-limiting hepatitis
Neurological complications	Yes	Yes
Deaths in pregnant females	Yes; 20–25% in final trimester^b^	No
Outcome in patients with underlying chronic liver disease	Poor	Poor
Chronic infection	No	Yes; genotype 3 only unknown
Burden of disease	3.4 million cases/yr, 70,000 deaths, 3,000 stillbirths	Unknown

a *HEV genotypes 3 and 4 have also been transmitted from human to human via infected blood products*.

b *The epidemiology and clinical course of HEV genotype 1 in Egypt are significantly different from those in other developing countries. In Egypt, the seroprevalence is similar to that of HAV, with nearly universal exposure in childhood, and the risks to pregnant females may be less. The reason for these observations is unknown*.

*^c^Data are for 9 of 21 regions defined for the Global Burden of Diseases, Injuries and Risk Factors Study (37), which represent 71% of the world's population*.

#### Mode of Transmission

##### Developing Countries

In underdeveloped countries, hepatitis E causes big epidemics of jaundice. These resource-poor regions have sources of drinking water contaminated from the sewers. HEV represent an important health issue in these endemic regions because it cause a huge number of cases and a significant number of deaths (4). The HEV-1 and HEV-2 genotypes predominate here and mainly infect humans, beeing transmitted through polluted water. Fecal elimination of the virus by people with clinical or subclinical forms of infection keeps a circulating conglomerate of infectious individuals that contaminates the water supply, resulting in the maintenance of the disease in endemic form. Virus detection in sewage systems shows a crucial role for an environmental reservoir (60, 61).

Throughout the year occur sporadic cases of hepatitis and occasionally large outbreaks occur, involving thousands of cases. In endemic areas, hepatitis E is about 30–70% of all cases of sporadic viral hepatitis. The incidence of the disease has been estimated to be around 45/1,000 people/year and, in India, it infects about 2.2 million persons/ year (4). Hepatitis E determine a self-limiting disease in most patients that lasts for several weeks, with the exception of pregnant women and people with underlying chronic liver disease ho have a high mortality rate. To date, limited studies have been performed that have failed to document chronic infection with genotypes 1 and 2.

Seroprevalence studies for anti-HEV IgG show that in many developing countries most children under the age of 10 have not been exposed to the virus. Between 15 and 30 years seroprevalence greatly increases and can reach about 30%. Exceptions to these results are in Egypt, where the seroprevalence of the disease is generally higher (up to 80%). Some studies show that the percentage of children exposed to the virus under the age of 10 is high (62). Seroprevalence studies performed on the South American population show varied results, between 1 and 20%. There are differences in terms of seroprevalence between and within countries, this reflecting the type of populations studied and the sensitivity of the methods that were used. In addition, hepatitis E has been studied in more detail in some countries, such as Argentina and Brazil, compared with others. HEV is extremely common in some countries from Southeast Asia (Pakistan, India, Bangladesh, and Nepal) and there have also been many major outbreaks in refugee camps from Africa over the past 20 years (63).

##### Developed Countries

Compared to poorly developed countries, in developed countries, most cases of HEV infection are acquired locally (domestic), and has become very clear the importance of animal reservoirs. HEV infection has become a real problem in Europe, North America, Japan and New Zealand, where HEV strains that infect mammals such as domestic pigs, wild boars, rabbits and deer are determinants agents of human zoonotic infection. The main animal reservoir of virus is represented by domestic pigs, in which the infection is asymptomatic; in many countries it has a very high prevalence. HEV-3 is spread all over the globe. In contrast, the HEV-4 genotype is widespread in China and Japan, but has recently been discovered in Europe in both pigs and humans. Genotype 3 and 4 have also been found in wild boars and deer, but the prevalence of these two genotypes is reduce compared to that found in domestic pigs (4).

It is not known exactly until now the full range of mammalian species that can be reservoirs for HEV. However, virus variants that infects rats, mongooses, ferrets, and bats have not yet been identified in humans. Mainly, the zoonotic transmission of the E virus can be done by eating uncooked or undercooked infected pork meat or game meat (rabbit, dear, and wild boar), raw liver from supermarkets and sausages intermediates (4).

The behavior of the virus in temperature changes and thermal stability was studied. It remains viable for 1 h after heating at 56°C. To fully inactivate the virus is required a cooking temperatures of 71°C for 20 min (64). HEV remains relatively stable in acidic and slightly alkaline environment and is not affected by treatment with ether and chloroform. HEV is sensitive to chlorine disinfection and it is recommended that during disease outbreaks to be done chlorine disinfection of water supply (65, 66).

Another possible route of transmission of HEV is by direct contact with HEV-infected animals. Many seroprevalence studies show that swine handlers and veterinarians have a higher risk of having anti-HEV IgG antibodies than the general population. Regarding genotypes 3 and 4, infection through water can also be important. HEV-3 has been detected in storage facilities for pig sludge, swine manure, untreated wastewater, river water, and also in oysters and mussels (67). In addition, it has been identified in several countries HEV infection transmitted by transfusion, and the virus has been identified also in blood products. For example, it was found that in donors from England, a total of 0.7% of small plasma bags contained HEV RNA and in donors from Germany was found a prevalence of 10% (68). The current estimates for the number of positive blood donations for HEV RNA are as follows: 1 from 7,040 in the UK, 1 from 4,525 in Germany, 1 from 3,090 in the Netherlands, and 1 from 1,430 in China (69, 70).

A very important aspect is to guarantee the effectiveness of the manufacturing process in inactivation/elimination HEV from biopharmaceuticals, such as medicinal products plasma-derived. The need for screening the donors for HEV RNA is discussed. In developed countries, seroprevalence studies for anti-HEV IgG are difficult. The results of many previous studies have shown very low seroprevalences, in the range of 1–2% (71, 72). The explanation may be that most of these studies used tests with low anti-HEV sensitivity so that true seroprevalence was more than likely significantly underestimated.

Several types of HEV transmission have been recognized.

##### Waterborne Transmission

In developing countries the spread of HEV is achieved through sewage water (6). From one epidemic to another, the way water is contaminated may differ. There are several possible situations in the environment: overcrowding with polluted water sources as in fast growing slum and refugee dwellings, plentiful monsoon rains; floods causing stagnation and reversal of flow in waterways polluting drinking water sources; seeping pipes supplying drinking water crossing across the sewage channels or sewage that flows into open drinking water sources (rivers and unprotected fountains) (58).

##### Person-to-Person Transmission

Outbreaks of hepatitis E in which the source of the infection is not identified are caused by person-to-person transmission. It is known that sporadic infections are spread among people (59).

##### Zoonotic Transmission

Studies have been performed on the prevalence of HEV among several animals in India. The infection has been present in several species of animals, including goats, sheep and buffalo and domestic pigs (73). All viruses identified in domestic pigs had the HEV-4 genotype in India. Regarding infections with human hepatitis E viruses, both epidemic and sporadic viruses are determined by the genotype 1. The opinion is that in this country the zoonotic transmission is insignificant in human infections (74).

In developed countries, genotypes HEV-3 and 4 are spread by zoonotic transmission through food, eating uncooked or undercooked flesh or liver from domestic pigs, wild boar, or Sika deer. Another way is through the wastewater of domestic pig manure that pollutes the navigable waters and infects people who walk the sea beach or eat contaminated seafood (75, 76).

##### Transfusion-Associated HEV

In several countries have been reported a number of cases and series of cases of transfusion-associated HEV infections (59, 60, 77). Many clinical conditions requires blood or blood products transfusions, in which hepatitis E is more severe or causes chronic hepatitis and cirrhosis such as pregnancy, liver illness, hematological neoplasm, solid organ transplantation, and HIV infected patients. Given the existing data, it is necessary to screen blood donors in countries with a high prevalence of HEV (59, 60).

##### Vertical Transmission

Several studies show that HEV can be transmitted vertically to the newborn from positive mothers with high maternal and fetal mortality (72, 78). Studies from literature show that especially in India, in certain geographical areas, in pregnant women, acute infection is associated with an increased risk of liver failure, and the mortality rate was up to 25% (72).

In pregnant women, infected with genotypes 1 and 2, excessive mortality rates are puzzling. There have been few documented cases of infections with genotypes 3 and 4, but they are generally not seen. The fetuses and newborns of mothers with acute infection in the third trimester have a 50 and 100% risk of infection in endemic regions, because the placenta acts as a viral reservoir. It was found in a recent study that in developing countries E virus infection may be responsible for >3,000 stillbirths each year, including fetal deaths due to maternal prenatal mortality. There are no clear data on the vertical transmission of HEV in non-endemic regions. The results of studies performed on a small number of patients in Southwest Europe suggest that this risk is low (72). Studies have been performed on the milk of infected mothers (colostrum) for HEV RNA and antibodies and lower levels have been found than in the corresponding serum samples. Studies have shown that children breastfed by infected mothers do not appear to have a higher risk than those who were artificially fed. To determine whether breastfeeding may be recommended for mothers infected with HEV further studies are needed (79).

#### HEV Pathogenesis

Although thoroughly studied, the pathogenesis of hepatitis E is still a mystery to the scientific world. Being known to have a mainly a fecal-oral transmission route, nobody has ever established how the virus finally gets to the liver. A hepatic site where the virus replicates might be identified, thus HEV replicating in the hepatocytes cytoplasm and being afterwards released both into bile and blood. HEV not being cytopathic, the liver may suffer from damages induced by HEV infection which are immune mediated by natural killer (NK) cells and cytotoxic T cells (4, 80).

Very important in transporting the lipids into plasma is the role played by Apolipoprotein E (ApoE) which also influences the release of viruses such as HCV (81) and their propagation. ApoE may be identified in the lipid membrane related to HEV virions, but also in the blood, thus contributing to the virus entry (33). In addition, the immune response to HEV may be modulated by ApoE through regulating the T lymphocyte proliferation and activation (82). Such association generally reveals various lifestyle habits, such as pork consumption (83).

On healthy controls, patients with acute hepatitis E also showed higher concentrations of IL-10 and higher proportions of CD25+CD4+ forkhead box P3+ (FoxP3+) regulatory T cells and a signature cytokine of regulatory T cells, all these details suggesting that these cells are of major importance in the disease acute phase. Though, further investigation of their role is highly required (84). Many studies have been made over pregnant women who although living in developing countries, were infected with HEV-1 which may have been the cause of a significant maternal mortality (30%, with most deaths in the third trimester), but there is still much uncertainty over this subject. The poorer results of the study led among pregnant women may be explained by HEV genotype, taking into account that HEV-3 is not specifically deadly for pregnant women (85, 86). As regarding the HEV-4 infections in pregnancy, no significant data has been provided yet.

Once with the pregnancy, important changes in the immune system and in the level of sex hormones occur, all being related to fetus protection against the mother's immune system (87). Hormones specific to pregnancy may lead to unwanted results, since pregnant women who test HIV-positive usually have higher levels of progesterone, estrogen and β-human chorionic gonadotropin compared to those who test negative to HIV (88). An *in vitro* study revealed that serum collected from pregnant women, mainly those being in the 3rd trimester, improved HEV replication through inhibiting type I interferon (IFN) expression and estrogen receptor. However, other studies scarcely proved that HEV can infect women who have high levels of HEV RNA (89–91).

#### Viral Prevalence

In order to make a consensus classification according to the complete genome sequences of HEV isolates, a viral taxonomic scheme was brought up recently. Thus, the *Hepeviridae* family consists of two genera: *Piscihepevirus* and *Orthohepevirus* (92, 93).

The main route of infection with HEV in humans is the oral one; afterwards, the virus may be excreted in their feces (94). The main causes of hepatitis E outbreaks in humans are heavy rains causing floods and the consumption of water which is contaminated with feces. Acute infection is generally induced by classical HEV while chronic hepatitis E appears only in vulnerable immunosuppressed patients at risk (95, 96). The incubation period for the clinical symptoms of acute hepatitis E to occur is from 2 to 6 weeks (97, 98). The main symptoms of hepatitis E are: nausea, fever, vomiting, abdominal pain, loss of appetite, itching, hepatomegaly, jaundice, pale stools and darkened urine. As epidemiological studies stated, the incidence of hepatitis E mainly appears in young adults (15–45 years old) (99–101) while the general mortality rate is ranged to 2% (102, 103) being able to grow above 20% in pregnant women from some areas, caused by fulminant hepatic failure (104, 105).

##### HEV Entry

There are two ways that HEV can be found: either as a non-enveloped virus, with the capsid interacting with the surrounding environment, or as a quasi-enveloped virus (eHEV) with the capsid coated in an exosomal membrane (106). Despite both forms being highly infectious, quasi-coated viruses are 10 times less infectious than uncovered viruses (107, 108).

Cell culture supernatant and serum-derived HEV was noticed to be much lower than urine-derived and fecal HEV, indicating the presence of quasi-covered and uncovered HEV both in the first and last type of samples (107, 109).

The capsid wraps the viral nucleic acid and the capsid itself is covered by another membrane which helps the virus bind and enter the host cell and also protects the viral genome from being environmentally damaged. The membrane must be thick enough to act as a protection shield for the nucleic acid, but it should also not be so seriously stable as to be able to undergo a change in its conformation when getting in contact with a host cell so that it enters the cell more easily and allow the viral genome to be released for replication. As far as enveloped viruses are concerned, the cellular uptake step is quite well-characterized. It begins with the virus attaching to the cell surface, resulting in recruiting some factors which are necessary for viral uptake by receptor mediated endocytosis. Nevertheless, the introduction process of non-enveloped viruses may be significantly different.

Although being similar to some factors at the site of entry in the initial stage of cellular uptake, as regarding the non-enveloped virus, its membrane fusion does not happen. In order to penetrate the outer membrane, a change of the conformational core protein is needed in these viruses. To establish an ongoing infection, high infection multiplicity is needed. Endocytosis is the main mechanism of absorption for most of the viruses and it contains clathrin-mediated endocytosis (CME), macropinocytosis, and caveolae-mediated endocytosis. Clathrin aggregates in the cytoplasm of the membrane as main characteristic of CME and is able to induce the formation of endosomes and of invagination. Due to the fact that HEV can be found either as quasi-coated HEV or as uncovered HEV, it is highly uncertain if the two forms of virus are actually internalized through the same mechanism (107, 110).

The uncovered HEV registration form and the eHEV entry continue by undergoing a different kinetic process. Accordingly, almost covered HEV a slower kinetic input compared to the uncovered HEV. One study showed that 90% of the maximum binding of uncovered particles was achieved in only 3 h after infection, while eHEV needed 6 h. In addition, the binding efficiency for naked HEV was 10 times higher than that of eHEV, thus resulting in a 2.7-times higher infectivity oh HEV. By using inhibitory molecules and RNA methods for depleting caveolin and clathrin, further investigation was performed over the mechanism of entry. The tests performed indicated that the caveolin blocking does not reduce the infectivity of eHEV and HEV, the two viral forms being actually inhibited by the clathrin blockade.

##### HEV Replication

HEV lacks an animal model but also an adequate *in vitro* culture system, thus the life cycle of HEV being hardly studied (111). The transmission route to the host is by entering the gut epithelial cells. The entire process supposes attaching to the surface of hepatocytes through heparin sulfate proteoglycans, binding to the receptor and finally entering the hepatocyte cells (112). Once incorporated into the cell, uncoating and releasing of RNA follows and, than, the translation into the non-structural proteins of the virus. In order to reproduce positive viral RNA in the negative direction of RNA, RNA-dependent polymerase is being used. As template for 2.2 kb subgenocmic RNA and 7.2 kb positive-sense RNA is being used the negative sense RNA. Subsequently, pORF3 and pORF2 were formed by the help of subgenomic RNA as a model. While genomic RNA and PORF2 protein are assembled into the new virion, the virus replication is optimized by pORF3. A lipid layer and pORF3 cover the virion resulting from the hepatocytes. Both the lipid synt layer and the PORF3 layer are further separated from the virion after getting out of the hepatocytes (113).

Two different RNAs are synthesized into subgenomic RNA of 2.2 kb and full-length genomic RNA (114). ORF1 translation has as template the genomic RNA which is packed into viral particles. The gemonic RNA also represent a template for the synthesis of additional negative strand RNA. In return, the ORF3 protein (13 kDa) and the capsid protein (72 kDa) have as template for translation the sub genomic RNA. ORF1 is only directly translated from the viral RNA; nevertheless, it is still uncertain if it acts as a multi-domain polyprotein or if it should be cleaved into to obtain and thrombin are important for viral replication and processing of ORF1.

Zinc salt application mainly affects HEV replication by directly inhibiting the RNA-dependent RNA polymerase (RdRp) and is useful in improving specific antiviral therapies (33).

In the junction region between ORF2 and ORF1 a highly conserved and promoted stem loop structure can be noticed for a capped bicistronic subgenomic RNA. ORF 3 and ORF2 proteins are encoded by it. The researchers investigated neither the functions of prolonged RNA which replicate the HEV in cell cultures or in infected individuals, nor their existence (115).

#### Clinical Course of HEV Infection

HEV infection has an incubation period of 2–6 weeks (116). Worldwide there are estimated 20 million HEV infections (117). There are four genotypes, genotype 1 and 2 infections are waterborne, while genotype HEV-3 and HEV-4 are zoonotic diseases. They are transmitted to humans through contact with pigs, food and blood transfusions. The main site of infection is the liver, also the small intestine, the colon, and lymph nodes (118). The HEV infection in humans may lead to acute or chronic hepatitis and extrahepatic manifestations. Acute illness can be caused mostly by genotypes 1 and 2 and it may lead to acute liver failure. The infection with HEV genotypes 1 and 2 was associated with high mortality rate in pregnant women (20%), which usually occurred in the third trimester (7, 11). The genotypes 3 and 4 have an asymptomatic course (only 5% are symptomatic) and are associated with usually liver failure only on patients with liver disease (119).

**Figure 1:**
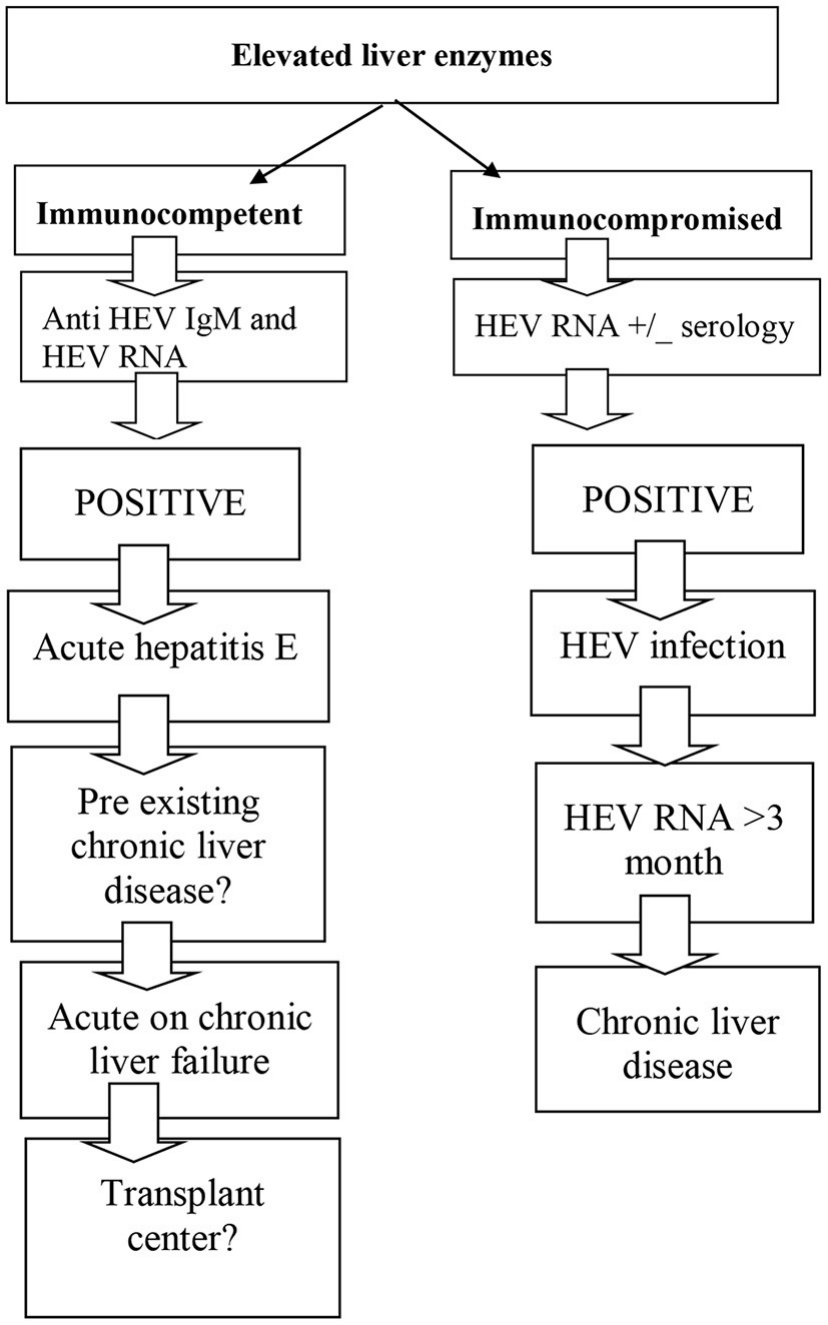
Diagnostic algorithm for HEV infection.

The algorithm of diagnosis in HEV infection is depicted in Figure 1.

The differential diagnosis of acute and chronic HEV infection is depicted in Table 3.

**Table 3:** Differential diagnosis of acute and chronic HEV infection.

**Differential diagnosis in HEV infection**	
Infection status	Differential diagnostic Drug induced liver injury
Acute infection	Auto imune hepatitis E B V hepatitis Acute hepatitis A Acute hepatitis B Acute hepatitis C CMV infection
Chronic infection into the immunosuppressed	Graft rejection Drug induced liver injury Intercurrent infection CMV or EBV reactivation

##### Acute HEV Infection

Studies reveal that the majority of symptomatic acute HEV infections are generated by genotype 1 and 2. The HEV genotypes 1 and 2 were responsible for mortality in 1% cases which involved immunocompetent adults often than young children (120). Acute HEV infection is generally asymptomatic or mildly symptomatic, 5–30% of symptomatic patients have icteric hepatitis (121). During the 1st week of HEV infection, the prodromal phase is characterized by nausea and vomiting, malaise, fever, and body aches. The prodromal phase is followed by icteric phase, characterized by dark colored urine and jaundice (121).

In patients who have no chronic liver disease, monitoring the liver function and enzymes, during the phase of acute infection, is sufficient. Even if progression to acute liver failure is rare, there are several studies that reveals a progression to acute liver failure (ALF) in 10% of patients (122). Genotype 3 and 4 seems to affect often male at age of 63, which are more susceptible to develop ALF (123). Studies analysis seems to reveal that this fact is related to host factors rather than differential viral expression. Studies revealed that ALF appears in 0.5–4% of patients with HEV infection. High mortality rate was associated with ALF, ~70% in most reports. The manifestation of ALF includes worsening of liver function with complications such as ascites, as encephalopathy and coagulopathy. In children, HEV clinical picture include fever, abdominal pain, vomiting, hepatomegaly. One study including 44 symptomatic children with HEV reveals that ALF appears in 15.9% (72, 122). Study analysis reveals that patients develop immunity against HEV once the infection is cleared but the re-infection is still possible.

##### Chronic HEV Infection

Chronic HEV infection is defined by HEV replication for more than 3 months (12, 36). HEV can cause chronic infection that can rapidly lead to cirrhosis (124). Several studies revealed that chronic HEV infection is diagnosed mostly in transplant population (18, 70, 124). Data suggest that in patients with SOT, children or adults, with HEV infection, which persists for over 3 months, chronic HEV infection must be considered. Studies showed that in patients with SOT, chronic HEV infection varies from 0.9 to 3.5% (70, 72, 124). Other studies revealed that HEV infection was detected among 4.3–6.5% transplant patients with high liver enzymes (18, 39, 76). It was reported that only 32% of SOT patients are symptomatic, the most common symptom reported was fatigue (18).

The liver fibrosis progression seems to be fast and it may lead to cirrhosis in 2–3 years after the infection. Only a few studies reveal incidents of infection in pediatric SOT, data suggest that HEV seroprevalence is 3.2%, and one in three children developed chronic HEV-3 infection (71, 72).

In immunocompromised individuals with HIV infection the rate of HEV infection was ranging from 1 to 3% (125, 126). In this group, data reveal that cirrhosis was observed in HEV-3 infection in individuals with low CD4 (<250/mm^3^) (126).

Few recent data give information about chronic HEV infection in immunocompromised children with SOT (3.2%) (70, 72). Some studies reported that only a small percentage (9.3%) of children with liver or combined liver-kidney transplant where tested HEV IgG positive but none of them was detected with chronic hepatitis (127).

##### Extrahepatic Manifestation

The main extrahepatic manifestations reported in patients with HEV infection are depicted in Table 4.

**Table 4:** The extrahepatic manifestations encountered in patients with HEV infection.

**System**	**Manifestation**
Neurological system	Guillain-Barré syndrome Neuralgic amyotrophy Encephalitis Myelitis Myositis Vestibular neuritis Peripheral neuropathy Bell's palsy Mononeuritis multiplex Seizure Pseudomotor cerebri Oculomotor palsy Polyradiculoneuropathy
Hematological system	Thrombocytopenia Monoclonal gammopathy of uncertain significance Hemolytic anemia Aplastic anemia Hemophagocytic syndrome CD(30+) cutaneous T cell lymphoproliferative disorder Thrombotic Thrombocytopenic purpura
Kidney	Relapse of IgA nephropathy Cryoglobulinemia Membranoproliferative glomerulonephritis
Heart	Myocarditis
Pancreas	Acute pancreatitis
Thyroid	Autoimmune thyroiditis Subacute thyroiditis
Skeletal system	Polyarthritis
Vasculitis	Henoch-Schönlein purpura

##### Neurological Manifestations

Several studies reported that most frequent neurological manifestation in children or adults with HEV infection is Guillain-Barre syndrome (GBS) and brachial neuritis (16, 17). GBS is a polyradiculoneuropathy which is presented as an evolving and ascending motor paralysis with hypotonia and areflexia, associated with an aleucocytic cerebrospinal fluid and elevated protein level. It is an autoimmune disease and it is triggered by bacterial and viral infection. There are two pathogenic mechanisms described by recent studies, one of the hypothesis raised the idea that GBS is induced by HEV virus which damages the nervous system directly by viral replication and the classic indirect immune mechanism of response (128).

We analyzed several studies that describe the association between HEV and GBS. GBS was developed frequently in patients with mild or moderate hepatitis symptoms for several days, which include vomiting, nausea, anorexia, fever, abdominal pain and hepatomegaly. GBS symptoms appeared meanly at 12 days after HEV infection. Most frequent symptoms were defined by weakness in the lower limbs that progressed to quadriplegia. Other symptoms included the triad of oculomotor weakness, areflexia and ataxia, bilateral lumbar poliradiculopathy, pure paraparesis, and acute sever midline back pain. The diagnostic of GBS was based on clinical presentation, positive results for IgM anti HEV antibodies and physical findings. Certain cases of HEV infection were confirmed by the presence of IgG anti HEV antibodies utilizing real time polimerase chain reaction (RT-PCR) for HEV in the serum (129–131).

##### Renal Manifestations

The renal disorders associated to HEV infection in children and adult patients include membrano-proliferative glomerulonephritis and cryoglobulinemia. Several studies observed a statistically decrease of the glomerular renal filtration rate (GFR) <5 ml/min during HEV infection (16, 132). Renal biopsies have been identified IgA nephropathy and membranoproliferative glomerulonephritis. In a large study 52% solid organ recipients patients presented cryoglobulinemia during chronic phase of infection and 36.4% during acute phase, compared to HEV negative solid organ recipients that associated crioglobulynemia in a lower percent (23.6%) (133).

##### Hematologic Manifestations

*Anemia*. The analyzed studies revealed that during HEV infection there were different patterns of anemia: hemolytic anemia, autoimmune hemolytic anemia, aplastic anemia. Hemolytic anemia was reported in 23% of patients with acute E hepatitis as a complication of acute infection (134). During viral infection, patients with glucose-6-phosphate dehydrogenase (G6PD) deficiency have lower level of glutathione, which leads to accumulation of oxidants and results in hemolysis during acute HEV infection (135, 136). Only a few cases of autoimmune hemolytic anemia were described in patients with HEV, on the basis on clinical manifestation, hypersplenism, and positive direct antiglobulin test (137, 138).

*Thrombocytopenia*. Thrombocytopenia can be induced, during HEV infection, by reduced hepatic production of thrombopoetin, hypersplenism, and bone marrow suppression by hepatotrophic viruses. It is mainly associated with HEV genotype 1 and 3. Usually, thrombocytopenia is self-limited (139).

*Acute pancreatitis*. In cases with HEV infection, most frequently acute pancreatitis is associated with non-fulminat hepatitis, and mortality depends on the severity of hepatitis. The published studies reported an average interval of 10 days between the jaundice appearance and acute pancreatitis onset (140, 141).

The researches revealed that acute pancreatitis was reported mainly among young males from endemic areas or among travelers to an endemic area. They usually develop mild to moderate pancreatitis (140). Also, complications of acute pancreatitis such as acute necrotizing pancreatitis, pseudocyst bleeding, and multiorgan failure where reported in several studies. The clinicians should consider acute pancreatitis comorbidity in cases of hepatitis E infection which associate severe abdominal pain. Mortality rate, caused by acute pancreatitis in patients with hepatitis E is 3.8%, similar to mortality rate reported in other causes of acute pancreatitis (142).

#### Diagnostic

Besides the characteristic clinical features described above, the evidence of HEV infection in pediatric and adult patients is based on several laboratory examinations. The lab tests implemented in the current diagnostic practice are based on specific antibodies detection such as immunoglobulin M (IgM) and immunoglobulin G IgG against VHE. The confirmation assay with a high accuracy is viral RNA detection using real time polymerase chain reaction (RT-PCR) for VHE genome. There are a lot of comparative studies regarding commercial vs. in-house assay for serologic antibodies detection. Evidence has been published describing different accuracy rates according to different VHE genotypes tested. Some test kits have a poor performance when applied in immuno-compromised pediatric or adult patients. The published data showed significant difference regarding the diagnostic accuracy of different testing methods. The reported sensitivity varied between 20 and 90% (143, 144). In clinical practice, the diagnosis of acute hepatitis with HEV among immunocompetent patients can be established with maximum precision by obtaining positive anti-HEV IgM antibodies results. At 14 days after the initial infectious contact with HEV, in 90% of cases IgM anti-HEV antibodies will be positive. Their level will remain detectable in serum up to 5 months (145).

In patients with associated immunodeficiencies, in the acute phase of HEV infection, it is recommended to assess the viral load of HEV RNA given the possibility of a low immune response in the synthesis of IgM antibodies against HEV (72, 144, 145).

The majority of available assays used in clinical practice are developed by sera from recent infected individuals. These test kits have lower accuracy compared to assays created and evaluated according to World Health Organization (WHO) reference reagents. The serological tests developed by using indirect enzyme-linked immunosorbent assay (ELISA) method have better sensitivity and specificity intermediates (146). The highest accuracy was reported for two asian marketed test-kits for IgM anti-HEV serum antibodies detection: Wantai Rapid test (Beijing Wantai Biological Pharmacy) and AssureTM (Genelabs Diagnostics, Singapore) (4, 145, 146).

Seroprevalence researches are based primarily on the determination of IgG antibodies against HEV. In cases with poor immune response in the synthesis of anti-HEV IgM antibodies, elevated levels of anti-HEV IgG antibodies support the diagnosis (147). Analysis of anti-HEV IgG antibodies is also essential in the following situations: screening of organ or blood donors considering the possibility of transmitting HEV through blood products, evaluation of the response to antiviral treatment and diagnosis of chronic HEV infection. Also of major importance is the analysis of IgG antibody level against HEV in order to evaluate the effectiveness of HEV vaccination. A serum antibody level equal or >2.5 WHO units/mL is considered protective after vaccination or acute infection (148).

In-house validates kits for viral load quantification of HEV RNA proved to have a lower accuracy compared to WHO standardized assays. HEV RNA can be assessed in blood and other fluids such as cerebrospinal fluid or ascites using primers based on conserved HEV genomic sequences. Loop mediated isothermal amplification (LAMP) is a reliable new technique using single step HEV RNA amplification (146, 149).

The most important studies on the pathogenesis of chronic HEV infection included post-transplant patients. The incidence of HEV comorbidity in these subjects varies between 0.9 and 3.5%, based on molecular testing—HEV RNA assesment (149, 150). Acute infection progressed into chronic phase in ~60% of patients (150). Other studies concerning the topic of seroconversion rate among patients with SOT have concluded that the progression to chronic phase varies between 21 and 50% (118, 151, 152).

A team of Belgian researchers assessed the accuracy of six serological tests and three PCR assays for HEV detection (153). Symptomatic patients with high suspicion of HEV infection were assessed for HEV IgM and IgG antibodies as well as for HEV molecular tests. Serologic screening was performed using six commercial HEV ELISA kits. HEV RNA was assessed using one commercial test kit and two optimized in-house real-time RT-PCR kit. Also, all three PCR tests were repeated on 16 external quality control samples. Seventy patients were enrolled. According to different ELISA tests, antibodies' prevalence was reported ranging between 5.7 and 14.3% for HEV IgM and 15.7–20.0% for HEV IgG. All PCR tests agreed these results, except two. Ten out of 16 control samples results showed major differences. The authors reported significant discrepancies regarding the accuracy of various serological and PCR tests, so they concluded that results of both serological and molecular tests for HEV should be interpreted with caution (153).

As shown previously, molecular tests have become essential tools for the diagnosis of immunocompromised patients associating chronic HEV infection. A team of researchers developed a new real-time reverse transcription-quantitative PCR (RT-qPCR) test utilizing Pleiades probe chemistry and an RNA internal control for the simultaneous detection and quantification of HEV RNA in human serum (154). Broadly reactive RT-qPCR primers targeting HEV ORF2/3 were successfully adapted for use in this assay, improving the detection and quantification of HEV RNA (154).

Table 5, adapted from Khuroo et al. (4), depicts the methods used for HEV diagnosis in different situation.

**Table 5:** The methods used for HEV diagnosis.

**Test**	**Method**	**Recommendation**	**Comments**
IgM anti-HEV	Enzyme-linked immunosorbent assay	Acute phase of infection	Different performance between assays lower sensitivity and sensibility in patients with immune disorders
	Immunochromatographic test		Concerning regarding diagnostic yield according to the genotype
IgG anti-HEV	Enzyme-linked immunosorbent assay immunochromatographic test	Seroprevalence studies chronic infection natural protection after acute infection serologic status post vaccination	Different performance between assays
HEV RNA	Real time polymerase chain reaction	Acute phase of infection confirm chronic phase of infection especially in immune-compromised subjects to evaluate anti-viral response organ or blood donors screening	Different performance between in house assays short-lasting viremia load present advantage/better accuracy when testing among subjects with immune conditions
HEV antigen	Enzyme immunoassay	Acute phase of infection	Relatively high accuracy 81% concordance with nucleic acid test HEV RNA

#### Prevention and Treatment of HEV Infection in Children and Adults

Control of epidemic outbreaks of HEV infection in underdeveloped countries is essential. Preventive measures must focus on ensuring clean sources of drinking water, correct and careful sewage disposal and compliance with general hygiene measures by the population. Chlorination of drinking water is also indicated to prevent the spread of HEV epidemics.

In the case of travel to countries with an increased incidence of HEV infection, it is recommended to avoid the consumption of unbottled water or sources of insufficiently cooked meat (fish, pork, etc.).

Due to the known risk of transmitting HEV infection through blood products, a thorough screening program of these products should be implemented before administration to patients from risk groups (155). Administration of hyperimmune immunoglobulin HEV, along with specific vaccination against HEV are important epidemiological measures in preventing the spread of HEV infection in children and adults (156).

In immunocompetent pediatric or adult patients with mild and moderate form of acute HEV infection, only symptomatic treatment is recommended due to the short-term evolution of the period with high viral load. In immunocompetent patients who develop severe forms of acute hepatitis with HEV, antiviral treatment with ribavirin for a period of 3 weeks is recommended. Initiation of antiviral therapy improves liver function and reduces serum transminase levels (4).

Ribavirin is known to have teratogenic effects, therefore its administration to pregnant women is debatable, despite the awareness of the risk of unfavorable maternal-fetal evolution in context of HEV infection in pregnant women.

The algorithm for treating chronic HEV infection has been assessed predominantly in immunocompromised pediatric or adult patients- especially those after solid organ transplantation or bone marrow transplantation, should avoid eating raw or undercooked meat, in order to decrease the risk of HEV contamination with genotype 3. Also, in post-transplant immunosuppressed patients infected with HEV, it is recommended to reduce the doses of immunosuppressive drugs in order to limit viral replication. Dose reduction of immunosuppressive drugs (mainly agents targeting T lymphocytes) used after solid organ transplantation, caused a significant decrease in HEV viremia in more than 30% of patients (157).

mTOR inhibitors and calcineurin inhibitors (tacrolimus and cyclosporine) stimulates viral replication and contributes to HEV persistence in the host organism. On the other hand, mycophenolate mofetil inhibits HEV viral replication and stimulates viral clearance in patients with chronic HEV hepatitis. If viremia persists for more than 3 months, it is recommended to initiate therapy with Ribavirin, which is the first-line antiviral (158). Another studied antiviral drug is pegylated α interferon, which is recommended for use in patients infected with HEV after liver transplantation, but not in patients with other types of solid organ transplants (159).

Sofosbuvir is a nucleotide analog polymerase inhibitor of hepatitis C virus. Several studies reported that this drug inhibits the viral RNA-dependent RNA polymerase, contributing to the reduction of HEV viremia level (160). The benefit of Sofosbuvir administration in different categories of patients with acute or chronic hepatitis with HEV should be evaluated in further clinical trials. Sustained viral response is defined by obtaining an undetectable value of HEV RNA viral load at 6 months after completion of antiviral therapy.

Treatment recommendations for chronic HEV infection in immunocompromised patients after solid organ transplantation are depicted in Table 6.

**Table 6:** Treatment recommendations proposed for chronic HEV patients after solid organ transplantation.

**Therapeutic option**	**The impact on viral replication**	**Recommendations**
mTOR inhibitors (everolimus, rapamycin)	Accelerates viral replication and increase the viremia level	Dose reduction
Calcineurin inhibitors (tacrolimus, cyclosporine)	Accelerates viral replication and promotes HEV persistence	Dose reduction
Guanosine analog (Ribavirin)	Reduces HEV multiplication and stimulates viral clearance	First line therapy
Cytokines (Pegylated α-Interferon)	Reduces HEV multiplication and stimulates viral clearance	Second-line therapy for cases non-responsive to ribavirin
Nucleotide analog (Sofosbuvir)	Block HEV multiplication *in vitro*	There are no current recommendations further clinical trials needed

The recommendations are based on data reported in case series reports. There are not large cohort studied in randomized clinical trials. Three patients with liver transplant and one patient with hemodialysis (161) who received a renal allograft who developed chronic hepatitis with HEV were treated for 3 months with pegylated interferon 135 mg/week. A sustained viral response was observed in only three of the four patients. Another study demonstrated the effectiveness of pegylated interferon for 12 months in treating chronic hepatitis with HEV in post-liver transplant patients (161, 162).

Summarizing the published data, pegylated α interferon is not recommended to be administrated used following heart, kidney or lung transplantation, because this drug amplify the risk of acute rejection (12, 162, 163).

Several clinical trials have been conducted that have included both pediatric and adult patients, post-transplant immunosuppressed and infected with HEV. The studies reported encouraging results in the efficacy of Ribavirin monotherapy for viral clearance in these patients. Data are shown in Table 7 adapted after Kamar et al. (12).

**Table 7:** Efficacy of ribavirin monotherapy in immunosuppressed patients.

**References**	**Enrolled patients**	**The reason for immunosuppression**	**Administration period (months)**	**Patients with sustained viral response**
Alric et al. (160)	1	Hematological pathology	3	1
Chaillon et al. (161)	1	Heart transplantation	3	1
	1	Renal transplantation	3	1
	1	Liver transplantation	3	1
Hajji et al. (164)	1	Human immunodeficiency virus infection	3	1
Junge et al. (165)	1	Heart transplantation	6	1
Kamar et al. (166)	8	Renal transplantation	3	4
Koning et al. (167)	4	Heart transplantation	3-12	3
Mallet et al. (168)	2	Simultaneous kidney/pancreas transplantation and hematological pathology	3	2
Neukam et al. (169)	1	Human immunodeficiency virus infection	6	1
Pischke et al. (155)	11	Renal, heart and lung transplantation	5	9
Pischke et al. (152)	4	Heart transplantation	5	3
Riezebos-Brilman et al. (170)	2	Lung transplantation	4	2

Only few published studies described several cases of HIV/HEV co-infected patients receiving antiviral therapy. A total of five patients received antiviral therapy. One patient received ribavirin monotherapy for 3 months (164, 165, 167) and two patients received ribavirin monotherapy for 6 months (166, 168, 169). One patient have been given for a period of 6 months pegylated interferon monotherapy followed by a period of 3 months of combined treatment with pegylated interferon and ribavirin (152, 170). One patient was treated for a period of 6 months with pegylated interferon monotherapy (171). Except for the patient who received 6 months of ribavirin monotherapy, a sustained viral response was obtained in all other patients (170, 171).

In patients with hematological disorders and chronic hepatitis with HEV, a sustained viral response was obtained according to published data, both after 3 months of monotherapy with Ribavirin and after a 3-month course of pegylated Interferon monotherapy (172, 173).

Extrapolating the data published from studies in the adult population, most pediatric patients with self-limiting forms of acute hepatitis with HEV do not need antiviral treatment, only supportive therapy (174, 175).

The treatment of acute liver failure in a child with HEV infection is supportive, including according to the evolution the need for liver transplantation. According to published pediatric studies, in acute hepatitis with fulminant HEV, short-term treatment with Ribavirin has been shown to be beneficial, reducing the need for liver transplantation (176, 177).

A short course of Ribavirin antiviral therapy is recommended for pediatric patients with pre-existing chronic liver disease who develop acute hepatitis with HEV (178). According to published studies, it is recommended to reduce the doses of immunosuppressive medication in immunocompromised children who develop hepatitis with HEV. If in these cases viral clearance is not obtained in the first 3 months, the initiation of Ribavirin therapy at a dose of 15 mg/kg/day for a period of at least 3 months is recommended. In order to monitor the viral clearance, the VHE RNA viral load will be determined monthly and depending on the evolution, the Ribavirin administration period may be extended (72).

##### Hev Vaccination

In order to reduce the incidence of HEV infection both among the population of underdeveloped countries and in rich, economically developed countries, it is necessary to implement a vaccination program based on a pan-genotypic HEV vaccine, especially for the population with associated risk factors.

A specific structural protein of HEV viral capsid represents the antigen against which most neutralizing antibodies are synthesized. Most experimental HEV vaccines are based on this primary target for inducing the immune response. More than 10 types of HEV vaccines have been assessed in phase one and two concerning laboratory and animal studies (primates). But so far, only two types of recombinant anti-HEV vaccines have been assessed in clinical trials on human subjects and launched on the market. For both vaccines there is a recommendation for three doses administration at start, after 1 and 6 months (179).

One marketed vaccine was obtained by biotechnology based on viral proteins derived from the structure of HEV expressed by a viral vector—baculovirus. The protein has 56 kDa molecular wight, it is encoded by ORF 2 sequence of HEV-1 genome and it is expressed by insect cells in order to produce the vaccine (4).

The study of the effectiveness of this vaccine compared to placebo showed a considerable immune response. Eighty one percent presented significant antibodies serum level within 1 month after the second dose and 100% of subjects developed protection 1 month after the third dose. No significant adverse effects have been reported. Following administration of all three vaccine doses, three cases of acute hepatitis with HEV were reported in the studied group of vaccinated individuals, compared with 66 subjects in the placebo group who developed the infection (180).

Chinese researchers have developed and launched an HEV vaccine based on structural viral HEV proteins expressed by the vector *Escherichia coli*. The chinese vaccine named HEV 239 (Hecolin) is represented by a protein with a molecular weight of 26 kDa encoded by sequence ORF2 of HEV-1 strain. It is expressed in *Escherichia coli*, resulting virus like particle of 23 nm in diameter. The effectiveness of the vaccine was tested in a placebo-controlled clinical trial that included more than 100,000 subjects aged 16–65 years. The immunological response quantified by antibody production was excellent in vaccinated subjects. One year after the third dose of HEV vaccines was administered, 15 cases of subjects infected with hepatitis E were detected in the placebo group, compared with zero cases in the group of vaccinated subjects (181). No serious adverse events were reported after vaccination. The post-vaccine antibody response was maintained at optimal parameters 4.5 years after the third dose administered to most subjects (182).

The vaccine developed by Chinese researchers has been shown to be effective in inducing protection against both the studied HEV 1 genotype and the HEV 4 genotype prevalent in the geographical area where the study was conducted, by cross-antibody response (4).

## Conclusions

HEV infection in children and adults is a condition with multiple polymorphic clinical features, with possible extrahepatic manifestations, which can in many cases remain underdiagnosed. Further large epidemiological studies are required to establish with certainty the true prevalence and incidence of this condition in both underdeveloped countries and rich countries especially among the adult and pediatric population at risk (post-transplant, immunosuppressed, etc.).

It is crucial to improve the management of acute and chronic hepatitis with HEV. In addition to studying new antiviral therapies, increasing prevention measures by assessing and implementation of specific HEV vaccination among vulnerable people (children, pregnant women, and immunosuppressed patients) is required.

## Author Contributions

OB, OA, AM, LO, and OM conceived and designed the study and wrote the paper. OB, EA, RF, AM, LS, LCS, and DG were involved in literature research, data collection, and data analysis. CZ reviewed and edited the manuscript. All authors contributed to the article and approved the submitted version.

## Conflict of Interest

The authors declare that the research was conducted in the absence of any commercial or financial relationships that could be construed as a potential conflict of interest.

## Publisher's Note

All claims expressed in this article are solely those of the authors and do not necessarily represent those of their affiliated organizations, or those of the publisher, the editors and the reviewers. Any product that may be evaluated in this article, or claim that may be made by its manufacturer, is not guaranteed or endorsed by the publisher.
